# An efficient and specific CRISPR-Cas9 genome editing system targeting soybean phytoene desaturase genes

**DOI:** 10.1186/s12896-022-00737-7

**Published:** 2022-02-15

**Authors:** Qing Shi Mimmie Lu, Lining Tian

**Affiliations:** Agriculture and Agri-Food Canada, London Research and Development Center, 1391 Sandford Street, London, ON N5V 4T3 Canada

**Keywords:** Genome editing, CRISPR/Cas9, Soybean, Stable transformation, Phytoene desaturase (PDS)

## Abstract

**Background:**

Genome editing by CRISPR/Cas9 has become a popular approach to induce targeted mutations for crop trait improvement. Soybean (*Glycine max* L. Merr.) is an economically important crop worldwide. Although gene editing has been demonstrated in soybean, its utilization in stably transformed plants through whole plant regeneration is still not widespread, largely due to difficulties with transformation or low mutation efficiencies.

**Results:**

We sought to establish a simple, efficient, and specific CRISPR/Cas9 system to induce heritable mutations in soybean through stable transformation. We targeted phytoene desaturase (PDS) genes due to the distinctive dwarf and albino phenotypes of the loss of function mutant. To evaluate gene editing efficiency and specificity, three constructs targeting each of the two homologous soybean PDS genes specifically, as well as two constructs targeting both simultaneously with one guide RNA were created. Instead of using cotyledonary nodes from germinated seedlings, we used ‘half-seed’ explants derived from imbibed seeds for *Agrobacterium*-mediated transformation of cultivar Williams 82. Transformed plants for all five constructs were recovered. Dwarf and albino phenotypes were observed in transgenic plants harboring the constructs targeting both PDS genes. Gene editing at the desired loci was detected in the majority of T0 transgenic plants, with 75–100% mutation efficiencies. Indel frequencies varied widely among plants (3–100%), with those exhibiting visible mutant phenotypes showing higher frequencies (27–100%). Deletion was the predominant mutation type, although 1-nucleotide insertion was also observed. Constructs designed to target only one PDS gene did not induce mutation in the other homologous counterpart; and no mutation at several potential off-target loci was detected, indicating high editing specificity. Modifications in both PDS genes were transmitted to T1 progenies, including plants that were negative for transgene detection. Strong mutant phenotypes were also observed in T1 plants.

**Conclusions:**

Using simple constructs containing one guide RNA, we demonstrated efficient and specific CRISPR/Cas9-mediated mutagenesis in stably transformed soybean plants, and showed that the mutations could be inherited in progenies, even in plants that lost transgenes through segregation. The established system can be employed to edit other genes for soybean trait improvement.

**Supplementary Information:**

The online version contains supplementary material available at 10.1186/s12896-022-00737-7.

## Background

Genome editing using the clustered regularly interspaced short palindromic repeats/CRISPR-associated 9 (CRISPR/Cas9) system has emerged as a versatile tool to modify genes at precise locations. It was initially discovered as bacterial immune system against viruses and other foreign nucleic acids such as plasmids [1–5]. CRISPR refers to direct repeats in bacterial genome separated by short stretches of variable sequences called spacers derived from invading genetic material [1, 4, 6]. Cas genes are often located adjacent to CRISPR, which encode proteins with RuvC-like and HNH-like nuclease domains [5, 7–9]. Transcription of CRISPR locus generates a non-coding precursor RNA cleaved into short CRISPR RNAs (crRNAs), which direct the Cas proteins to cleave foreign nucleic acids containing complementary target sequences [2]. The Type II CRISPR system from *Streptococcus pyogenes* is among the best characterized. It is consisted of nuclease Cas9, crRNA, and an auxiliary trans-activating crRNA (tracrRNA) required for processing of crRNA into functioning units [7, 10]. In the engineered CRISPR/Cas9 system, crRNA and tracrRNA are fused into a single guide RNA (sgRNA) [7, 11]. Cas9 is guided to specific genomic locus by sgRNA containing a 20-nucleotide (nt) target sequence, which immediately precedes the protospacer adjacent motif (PAM), with the sequence 5’-NGG-3’ in the system derived from *Streptococcus pyogenes* [7, 11–13]. At the target site, Cas9 RuvC and HNH-like nuclease domains each cleave one DNA strand, creating a double stranded break (DSB) at 3 base pairs (bp) upstream of PAM [7, 9]. The cleaved genomic locus then activates DNA damage repair, either by nonhomologous end joining (NHEJ) pathway or homology-directed repair (HDR) [11–13]. In the absence of a repair template, the error prone NHEJ pathway is activated, creating insertion or deletion (indel) mutations. HDR pathway requires the presence of homologous DNA template surrounding the DSB, which can be delivered by a plasmid or single-stranded DNA oligos [12, 13]. Once mutations are induced at target loci, novel traits can be retained in transgene-free mutants through transient Cas9/sgRNA expression or Mendelian gene segregation. Genome editing using CRISPR/Cas9 has been employed in various plant species of commercial importance such as rice [13], wheat [13, 14], apple [15], tomato [16], grape [17, 18], melon [19], etc. This technology has become a promising tool for crop trait improvement.

Soybean (*Glycine max* L. Merr.) is a globally important crop that provides a rich source of protein and oil. Since year 2015, genome editing in soybean has been performed by several research groups, often using hairy root system [20–29]. Although hairy root system is less labour-intensive and time consuming compared to whole plant regeneration, targeted mutations are limited to root tissues, and cannot be inherited to subsequent generations. Therefore, this method is not suitable for agronomic trait improvement. To produce stable germplasm harboring desired mutations, it is important to employ the genome editing technology through whole plant genetic transformation. In more recent years, publications involving CRISPR/Cas9 in stably transformed soybean plants have emerged [21, 27, 29–38]. Most of these studies used cotyledonary nodes from newly germinated seedlings for *Agrobacterium*-mediated transformation [21, 27, 29, 33, 34, 36, 38]. Although successful gene editing has been demonstrated in stably transformed soybean plants, mutation efficiencies can be limited [29, 31, 36], and in some cases, few T0 transgenic plants were obtained [33, 35]. As a result, utilization of genome editing in soybean through whole plant transformation is still considered challenging [33, 39].

We sought to establish a simple, efficient, and specific system for CRISPR/Cas9-mediated mutagenesis in stably transformed soybean that can transmit the mutations and altered traits to progenies. We selected soybean phytoene desaturase (PDS) genes for editing through NHEJ, due to the distinctive phenotypes of the loss of function mutant. In Arabidopsis, PDS encodes an enzyme in the carotenoid biosynthesis pathway, disruption of this gene results dwarf and albino phenotypes [40], making it a widely used indicator for genome editing in plants [13, 15, 18, 19, 21, 37, 41–43]. Two homologous PDS genes are present in the soybean genome. Previously, Du et al. [21] targeted both genes in cultivar (cv.) Jack through stable transformation. Although mutations were induced in adventitious buds, fully regenerated T0 plants were not recovered. Very recently, Zhang et al. [37] also targeted both genes simultaneously, and obtained a good number of fully regenerated T0 plants showing gene editing. However, assessment of targeting specificity was not reported by both studies. Moreover, neither examined the inheritance of mutations beyond the T0 generation. To evaluate gene targeting efficiency and specificity, we created three constructs targeting each PDS gene specifically as well as two constructs targeting both genes simultaneously, using one guide RNA in each construct. Instead of using cotyledonary nodes derived from germinated seedlings, we performed *Agrobacterium*-mediated transformation of cv. Williams 82 using ‘half-seed’ explants dissected directly from overnight-imbibed seeds. Transformed plants for all constructs were recovered. Mutations at desired loci were induced in T0 plants with high specificity and efficiencies, and were transmitted to T1 progenies. Simultaneous targeting of both PDS genes resulted in visible mutant phenotypes in both T0 and T1 generations. Our efficient and specific CRISPR/Cas9 genome editing system can be employed to modify other genes in soybean through whole plant transformation for agronomic trait improvement.

## Results

**Fig. 1:**
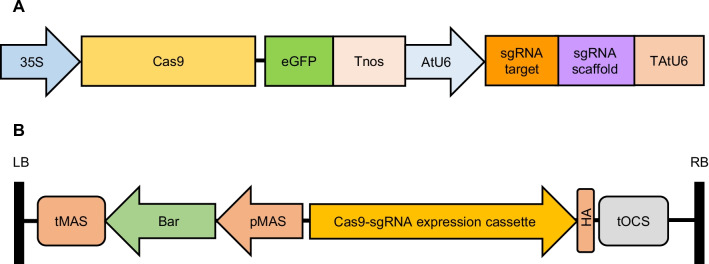
Schematic diagram of Cas9/sgRNA expression cassette and T-DNA region of the genome editing construct. **A** Cas9/sgRNA expression cassette contains a 35S promoter driving the expression of Cas9 translationally fused to eGFP, followed by nopaline synthase terminator (Tnos); as well as Arabidopsis U6 promoter (AtU6) driving the expression of sgRNA, followed by AtU6 terminator (TAtU6). The sgRNA is comprised of a scaffold for Cas-binding and 20-nt target sequence. **B** T-DNA region containing Cas9/sgRNA expression cassette in binary vector pEG301. LB, left border; RB, right border; tMAS, mannopine synthase terminator; Bar, phosphinothricin acetyltransferase conferring BASTA resistance; pMAS, mannopine synthase promoter; HA, human influenza hemagglutinin tag; tOCS, octopine synthase terminator

Soybean is a palaeopolyploid organism which underwent genome duplication in ancient times, nearly 75% of its genes are in multiple copies [44]. The soybean genome contains two highly homologous PDS genes: Glyma.11g253000 on chromosome 11 (hereafter named GmPDS11g) and Glyma.18g003900 on chromosome 18 (hereafter named GmPDS18g). The two paralogues have 13 exons in each, sharing 96% identity in nucleotide coding sequence (Additional file 1, Figure S1), and 98% identity at amino acid level (Additional file 2, Figure S2). We created an array of genome editing constructs, including two constructs (GmPDS1 and GmPDS3) targeting GmPDS18g specifically, one construct (GmPDS7) targeting GmPDS11g specifically, and two constructs (GmPDS8 and GmPDS9) targeting both genes simultaneously at conserved regions (Table 1). We used CRISPR-PLANT online tool [45] to select guide sequences targeting each gene specifically. Sequences targeting both PDS genes simultaneously were designed manually by selecting 20 nucleotides in the conserved regions immediately preceding PAM ‘-NGG-’, on exons in the upstream locations. The sequences were then used as queries for Basic Local Alignment Search Tool (BLAST) in Phytozome [46] to check for specificity. All sgRNA target sequences are in the sense orientation. Coincidentally, our GmPDS7 has the same guide RNA sequence as S11 in Du et al. [21], and GmPDS8 starts at 3-nt upstream of D7 in Du et al. [21] as well as the GmPDS guide RNA in Zhang et al. [37]. Each genome editing expression cassette contains a 35S promoter driving the expression of a maize codon optimized Cas9 [47, 48] translationally fused to green fluorescent protein (eGFP), as well as Arabidopsis AtU6 promoter driving the expression of sgRNA containing the 20-nt target sequence (Fig. 1A). The expression cassette was cloned into binary vector pEarleygate301 (pEG301), which confers resistance to herbicide BASTA (Fig. 1B). *Agrobacterium*-mediated genetic transformation was performed using ‘half-seed’ explants based on the methodology described by Paz et al. [49] to deliver the constructs into soybean cv. Williams 82, which was the genotype used to generate the reference genome sequence [44].

**Table 1:** Overview of the genome editing constructs targeting soybean PDS gene(s)

Construct	Target gene(s)	Target site	sgRNA target sequence (5'—3')
GmPDS1	GmPDS18g	exon 4	CCTAATGTGCAGAACCTTTT
GmPDS3	GmPDS18g	exon 6	ACCTGAACGGGTAACTGATG
GmPDS7	GmPDS11g	exon 4	GTCCTTCCCGCCCCATTAAA
GmPDS8	GmPDS11g & GmPDS18g	exon 2	CTGGAAGCAAGAGACGTTCT
GmPDS9	GmPDS11g & GmPDS18g	exon 5	TCAAGAATGGATGAAAAAGC

### T0 GmPDS1, GmPDS3, and GmPDS7 Plants are phenotypically similar to wild type

**Fig. 2:**
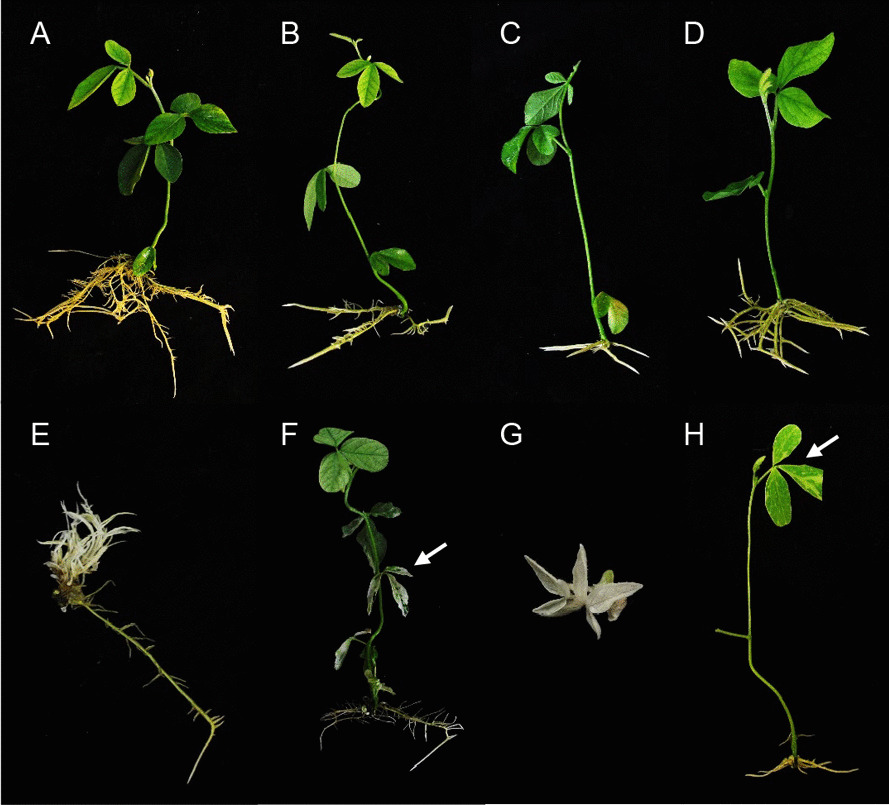
Phenotypes of soybean plants transformed with various genome editing constructs. **A** Wild type Williams 82 plantlet regenerated from tissue culture. **B** Regenerated GmPDS1 plantlet. **C** GmPDS3 plantlet. **D** GmPDS7 plantlet. **E** GmPDS8 plantlet showing strong dwarf and albino phenotypes. **F** GmPDS8 plantlet with variegated leaves (pointed by arrow). **G** Albino GmPDS9 shoot. **H** GmPDS9 plantlet with variegated leaves (pointed by arrow)

We recovered two plantlets from GmPDS1 and eight plantlets from GmPDS3 transformation procedures. To verify transformation, genomic DNA was extracted from leaf tissues and used for polymerase chain reaction (PCR) using construct-specific primers (Additional file 7: Table S5). A 885-bp fragment containing AtU6 promoter, sgRNA, AtU6 terminator, and part of pEG301 backbone was amplified. Example agarose gel showing the amplicon is in Additional file 8: Figure S3. Transgene was detected in two independent GmPDS1 plants as well as five GmPDS3 plants of four independent events. The majority of T0 plants came from different explants, denoted by different numbers. Those developed from the same explant are considered the same event, denoted by the same number followed by a different alphabet. Seven independent plantlets were recovered from GmPDS7 transformation. Four of which survived in soil, all had the transgene detected. All plants transformed with the three constructs were phenotypically similar to regenerated control Williams 82 plants. No dwarf or albino phenotype was observed (Fig. 2A–D).

### Mutations were detected in T0 GmPDS1, GmPDS3, and GmPDS7 plants, in the desired loci specifically

To analyze gene editing, we sequenced PDS gene fragments encompassing the target sites in GmPDS1, GmPDS3, and GmPDS7 T0 plants verified for transformation. To examine the presence of basal mutation in the regions, we also sequenced regenerated wild type controls, as well as several GmPDS3 plants that were negative for transgene detection. In all cases, gene fragments amplified from wild type plants matched the reference sequences. Plants that were negative for transgene detection also showed no sequence change (Additional file 4: Table S2), indicating absence of basal mutation in the loci. Mutations at target sites were detected in all GmPDS1 and GmPDS3 transgenic plants, with 100% mutation efficiencies. Sequence changes were also detected in three GmPDS7 transformants, corresponding to 75% mutation efficiency (Table 2).

**Table 2:** Summary of CRISPR/Cas9-induced mutations in T0 plants

Construct	# of sequenced plants	# of plants verified for transformation	# of plants with mutations in GmPDS18g	# of plants with mutations in GmPDS11g	Mutation efficiency (%)	Indel frequency (%)
GmPDS1	2	2	2	0	100	6.5–16
GmPDS3	8	5	5	0	100	8–88
GmPDS7	4	4	0	3	75	9–21
GmPDS8	11	10	9	9	90	3–100
GmPDS9	6	5	4	5	90	4–100

Mutation efficiency (%) is calculated as number of plants showing mutations divided by number of plants verified for transformation

Indel frequency (%) is calculated as number of clones showing mutations divided by total number of sequenced clones

For each transgenic plant, we sequenced both GmPDS11g and GmPDS18g regions. The target fragments were amplified from genomic DNA, cloned into plasmid pGateG [48], and transferred into *E.coli*. Plasmids were extracted from randomly selected *E.coli* colonies and sequenced. Of the two GmPDS1 plants, 6.5% and 16% of sequenced clones had mutations in GmPDS18g, these numbers represent indel frequencies at the target site. Likewise, the five transgenic GmPDS3 plants had indel frequencies of 8%, 12%, 23%, 74% and 88% in GmPDS18g (Table 2, Additional file 4: Table S2). On the other hand, no sequence change was detected in the homologous counterparts in GmPDS11g, providing evidence of specific targeting. Indels in GmPDS11g were detected in three GmPDS7 transformants, among 9%, 14%, and 21% of sequenced clones (Table 2, Additional file 4: Table S2). No mutation was detected in the GmPDS18g counterpart, again demonstrating gene targeting specificity.

**Fig. 3:**
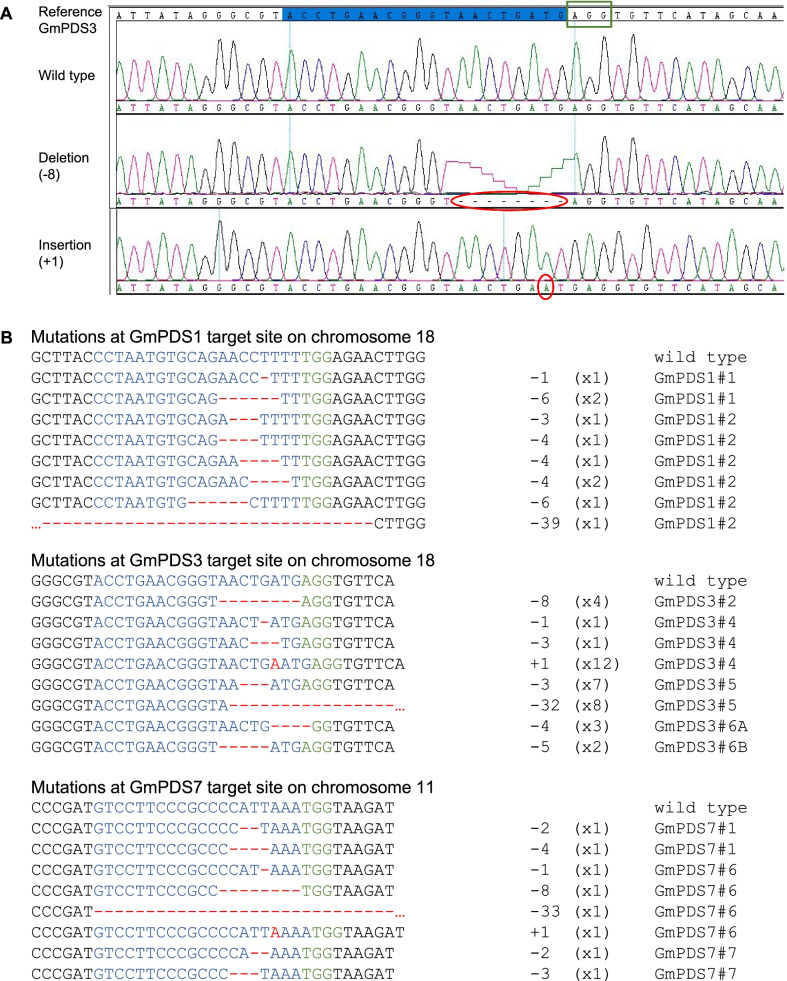
Detection of mutations in GmPDS1, GmPDS3, and GmPDS7 T0 plants. **A** Examples of sequencing chromatograms showing mutations. The 20-nt target sequence is highlighted in blue, PAM sequence is indicated in the green box, and mutations are circled in red. **B** Representative mutations at target sites. Individual T0 plant harboring the mutation is indicated to the right of each sequence. Wild type sequence is shown at the top with the 20-nt guide sequence in blue and PAM sequence in green. Mutations are shown in red. The number of mutated nucleotides is indicated to the right of each sequence. -: deletion, + : insertion. The number of clones for each mutation is indicated in brackets

The observed mutations predominantly occurred in the guide RNA regions, a few bp upstream of PAM. The vast majority were short deletions of one to several nucleotides (Fig. 3). Longer deletions of over 30 nucleotides were detected in some clones. One-nt insertion in front of PAM was also common. Insertions longer than 1-nt were not detected.

### Simultaneous targeting of both PDS genes at conserved regions resulted in dwarf and albino phenotypes

A total of 21 GmPDS8 T0 plantlets, of 17 independent events, were regenerated. Among those, 19 were confirmed for transformation by PCR. A range of phenotypes were observed: six plantlets were strongly dwarf and albino (Fig. 2E), typical of the *pds* loss of function mutant [40]; three were partially dwarf with albino or variegated leaves; three had pale leaves; one had variegated leaves, but not dwarf (Fig. 2F); and eight had phenotypes similar to wild type. Of the six shoots with strong mutant phenotypes, only three could form roots. Due to developmental defects, only two T0 plants with visible mutant phenotypes (line #3 and #5) survived in soil.

Fifteen plantlets of independent events were recovered from GmPDS9 transformation. Among those, one plantlet showed variegated leaves (Fig. 2H); three shoots were completely or partially albino that did not elongate and form roots (Fig. 2G); one shoot was pale green that failed to develop roots; and the ten remaining plantlets were phenotypically similar to wild type. Due to loss of plants growing in soil, only 11 plantlets were collected to verify transformation by PCR. Transformation efficiencies for all constructs are indicated in Additional file 3: Table S1, the highest being 7%.

### Mutations were detected in T0 GmPDS8 and GmPDS9 plants, in both PDS genes

We selected 11 GmPDS8 T0 plants for sequencing, of which 10 were positive for transgene detection. Two plants developed from the same explant (#6A, #6C), and the rest were all independent lines, denoted by different numbers. Mutations were detected in 9 out of 10 transgenic plants in both PDS genes, corresponding to 90% mutation efficiency (Table 2, Additional file 4: Table S2). Interestingly, plants with visible mutant phenotypes had higher proportions of clones that showed gene editing, compared to those with wild type phenotypes. Specifically, plant #6C exhibited strong dwarf and albino phenotypes and all sequenced clones had mutations at target loci in both PDS genes, equivalent to 100% indel frequencies. Plant #7 and #10 also developed strong mutant phenotypes, they had 70% and 61% indel frequencies in GmPDS18g, as well as 52% and 68% indel frequencies in GmPDS11g, respectively. Plant #3 and #12 were partially dwarf, with albino leaves; 46% and 60% of clones showed mutations in GmPDS18g, as well as 39% and 29% of clones with mutations in GmPDS11g, respectively. Plant #5 was not dwarf, but had variegated leaves. It had 65% and 64% indel frequencies in GmPDS18g and GmPDS11g, respectively. Other plants exhibiting wild type phenotypes had between 4 to 15% indel frequencies in GmPDS18g, and 3% to 13% in GmPDS11g.

We sequenced six independent GmPDS9 T0 plants. Out of the five plants verified for transformation, four had mutations in both PDS genes, and one had mutation only in GmPDS11g. The combined mutation frequency was 90% for both loci (Table 2). Similar to GmPDS8 plants, higher indel frequencies were observed in plants with strong mutant phenotypes (Additional file 4: Table S2). Plant #12 and #14 developed albino shoots that could not form roots. In both cases, mutations were detected in all sequenced clones for both PDS genes (100% indel frequencies). Interestingly, plant #12 had the same mutation in GmPDS18g, which is 1-nt insertion at 2-bp upstream of PAM (Fig. 5). Plant #5 and #15 had variegated and partially albino leaves. These 2 plants had 27% and 67% indel frequencies in GmPDS18g, as well as 85% and 88% indel frequencies in GmPDS11g, respectively. Similar to plant #12, plant #5 had the same 7-nt deletion a few bp upstream of PAM in GmPDS11g. Plant #6 exhibited phenotypes similar to wild type, only one clone showed a 2-nt deletion in GmPDS11g, while no mutation was detected in GmPDS18g.

Together, these findings demonstrate that a single guide sequence at conserved region can simultaneously induce mutations in both PDS genes, and the severity of mutant phenotypes relates to indel frequencies. Again, wild type controls as well as the GmPDS8 and GmPDS9 plants negative for transgene detection did not show sequence change in both PDS genes, indicating absence of confounding basal mutations (Additional file 4: Table S2).

**Fig. 4:**
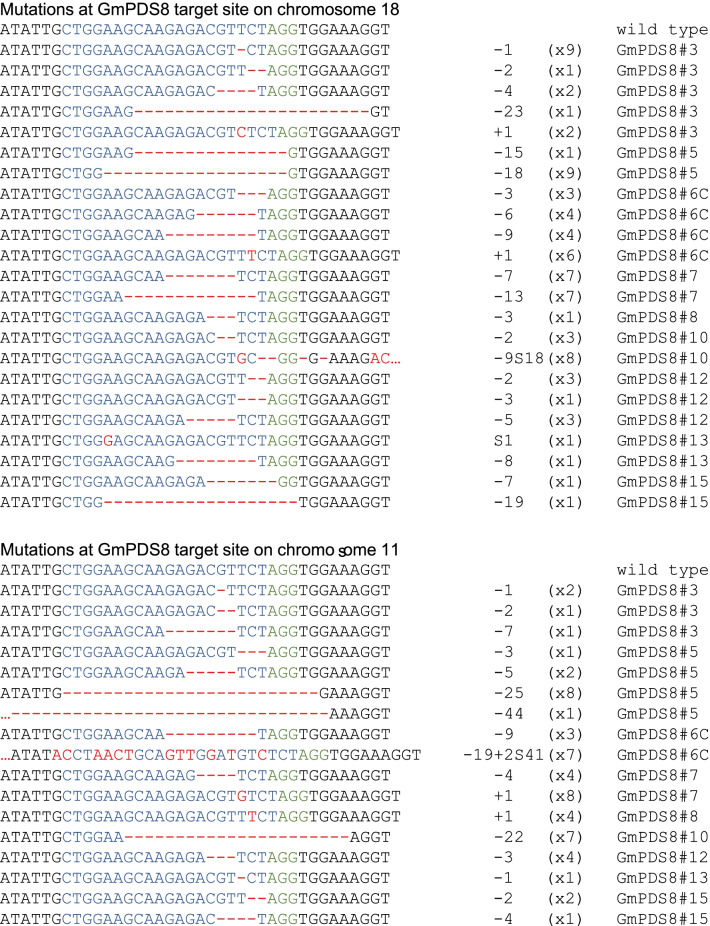
Representative mutations at target sites in GmPDS8 T0 plants. Individual plant harboring the mutation is indicated to the right of each sequence. Wild type sequence is shown at the top with the 20-nt guide sequence in blue and PAM sequence in green. Mutations are shown in red. The number of mutated nucleotides is indicated to the right of each sequence. -: deletion, + : insertion, S: substitution. The number of clones for each mutation is indicated in brackets

**Fig. 5:**
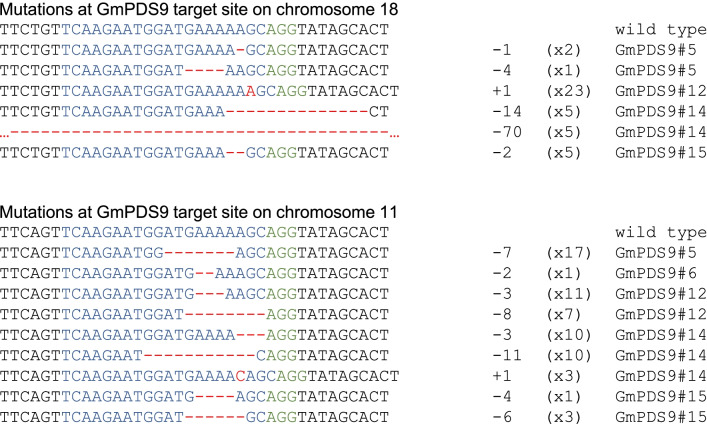
Representative mutations at target sites in GmPDS9 T0 plants. Individual plant harboring the mutation is indicated to the right of each sequence. Wild type sequence is shown at the top with the 20-nt guide sequence in blue and PAM sequence in green. Mutations are shown in red. The number of mutated nucleotides is indicated to the right of each sequence. -: deletion, + : insertion. The number of clones for each mutation is indicated in brackets

The majority of transgenic plants had multiple mutations at each target site. Both insertions and deletions were detected. Similar to plants transformed with other genome editing constructs, mutations in GmPDS8 and GmPDS9 T0 plants were predominantly small deletions within the guide RNA regions, occurring one to several nucleotides upstream of PAM (Fig. 4, 5). In some cases, large deletions of over 15-nt encompassing PAM were detected. One-nt insertions at 2–3 bp upstream of PAM were also common, although less frequent compared to deletions (Figs. 4, 5). In three GmPDS8 plants, 1-nt substitution, or a combination of mutation types were observed (Fig. 4).

### Simultaneous targeting of both PDS genes did not induce mutations in several potential off-target sites

To further examine the specificity of PDS gene targeting, we performed off-target analysis in two GmPDS8 T0 plants with visible mutant phenotypes: #5 and #6C. By using the 20-nt guide RNA sequence as query in BLAST search, potential off-target loci were identified in two homologous auxin response factor genes (Glyma.10g053500 and Glyma.13g140600) as well as two homologous GIGANTEA genes (Glyma.10g221500 and Glyma.20g170000). They are hereafter referred as ARF10, ARF13, GGT10, and GGT20, respectively. It is worth noting that all of these sites are followed by PAM ‘AGG’. ARF10 and ARF13 contain 5 and 10 consecutive mismatches to GmPDS8 target sequence, located immediately upstream of PAM. GGT10 and GGT20 have 7 and 6 interspaced mismatches, respectively; both contain 4 consecutive mismatches directly upstream of PAM (Additional file 6, Table S4). No mutation at those sites was detected (Additional file 6: table S4), indicating high specificity for PDS gene editing.

### CRISPR/Cas9-induced mutations and altered phenotypes were inherited in T1 generation

**Fig. 6:**
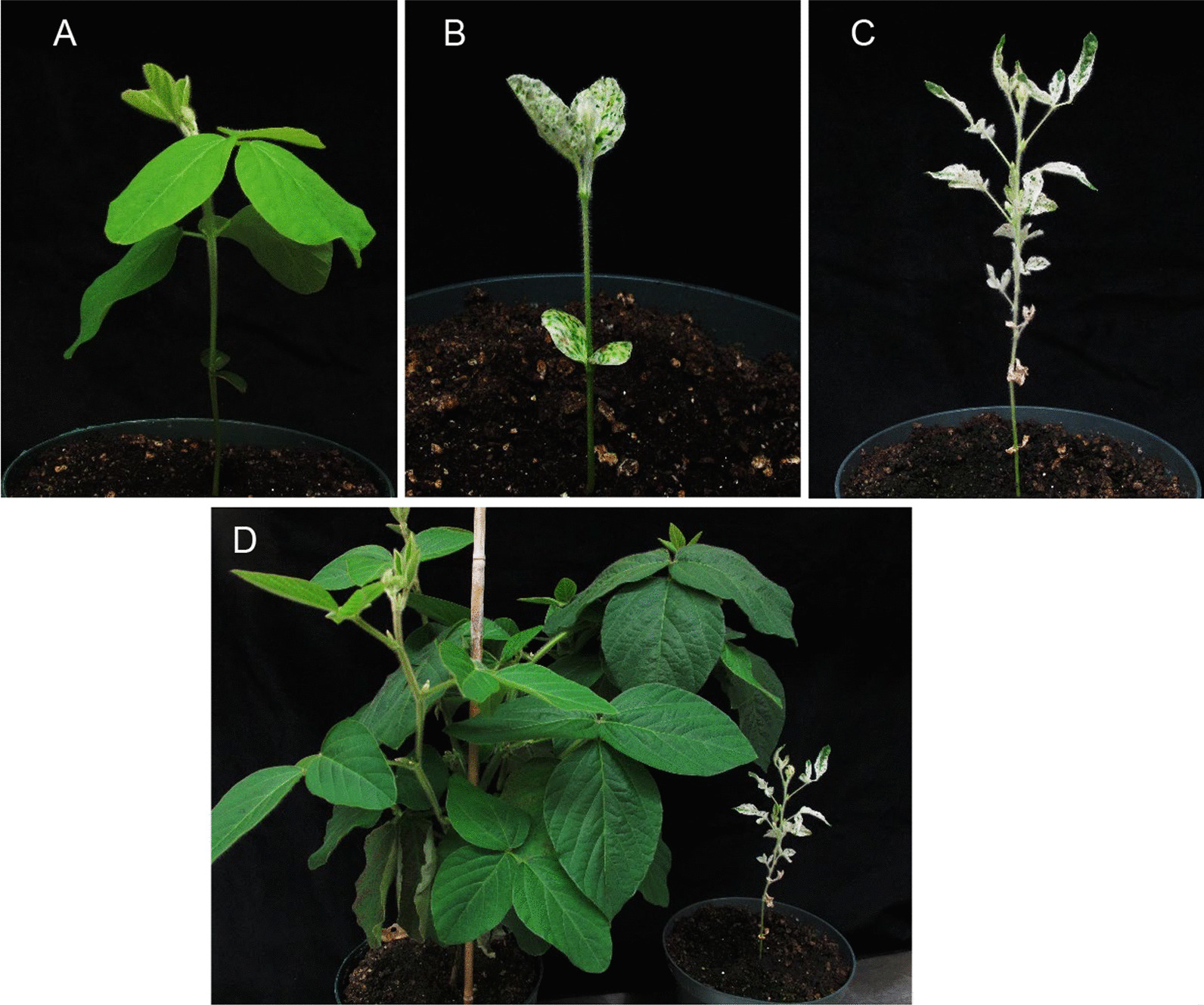
Phenotypes of GmPDS8 T1 plants derived from T0 plant #5. **A** GmPDS8 T1 plant at 3 weeks after germination, showing phenotypes indistinguishable from wild type. **B**, **C** GmPDS8 T1 plant showing dwarf and albino phenotypes at 3 weeks (**B**) and 2 months (**C**) after germination. **D** Side by side comparison between 2-month old T1 plants with wild type and mutant phenotypes

**Fig. 7:**
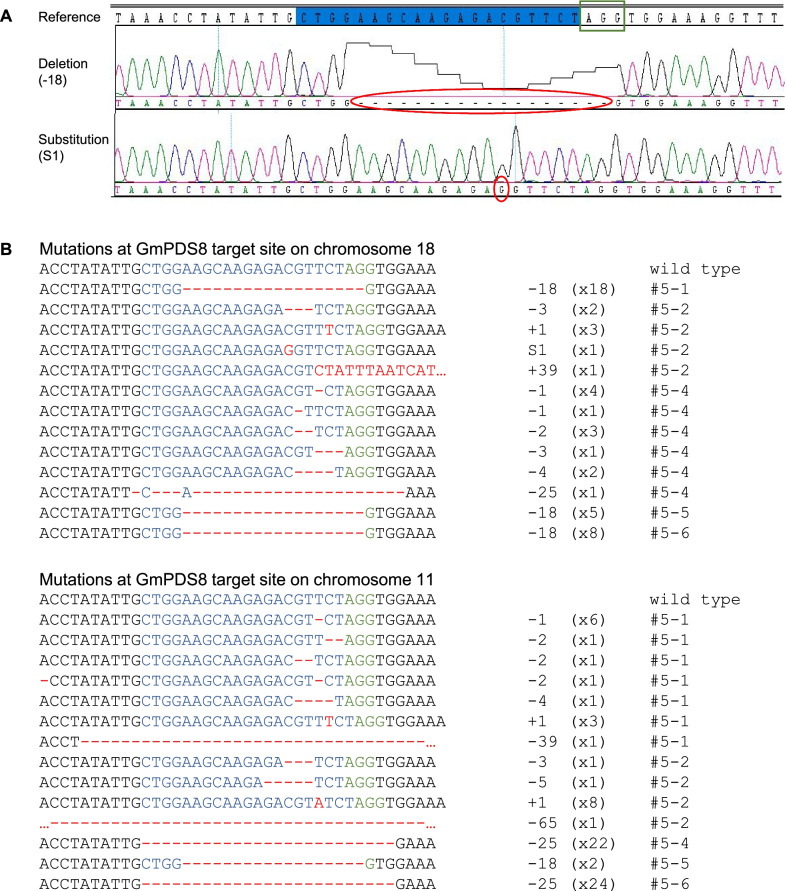
Detection of mutations in GmPDS8 T1 progenies derived from T0 plant #5. **A** Representative sequencing chromatograms showing mutations. The 20-nt target sequence is highlighted in blue, PAM sequence is indicated in the green box, and mutations are circled in red. **B** Representative mutations at target sites on chromosome 18 and chromosome 11. Individual T1 plant harboring the mutation is indicated to the right of each sequence. Wild type sequence is shown at the top with the 20-nt guide sequence in blue and PAM sequence in green. Mutations are shown in red. The number of mutated nucleotides is indicated to the right of each sequence. -: deletion, + : insertion, S: substitution. The number of clones for each mutation is indicated in brackets

To examine whether gene editing induced by CRISPR/Cas9 can be passed down to the next generation, T1 plants from three GmPDS8 lines were analyzed. Because of limited amount of seeds produced from T0 plants, all T1 seeds from GmPDS8 line #3 (1), #5 (7), #8 (2), #13 (5), and #15 (8) were planted in soil, with respective quantities for each line indicated in brackets. Due to developmental defects, seeds from line #3 and #8 did not germinate. A total of six T1 line #5 plants grew in soil, among which three (#5–1, 5–2, 5–4) exhibited strong dwarf and albino phenotypes, while others (#5–3, 5–5, 5–6) had phenotypes indistinguishable from wild type (Fig. 6). To detect the presence of transgene in this generation, PCR analysis was conducted using two sets of construct-specific primers (Additional file 7: Table S5). The first set amplified a 885-bp fragment described earlier. The second set amplified a 1117-bp fragment containing part of Cas9 and eGFP. The presence of transgene was only detected in the three plants with strong mutant phenotypes (Table 3, Additional file 8: Figure S3). Consistent with these findings, mutations were detected in all sequenced clones for both PDS genes in plant #5–2 and 5–4 (100% indel frequencies). Plant #5–1 had 100% and 93% indel frequencies in GmPDS18g and GmPDS11g, respectively (Table 3, Additional file 5: Table S3). Similar to the T0 generation, the vast majority were small deletions upstream of PAM. Longer deletions of up to 65 nucleotides were observed as well. One-nt insertions were also detected. More rarely, 1-nt substitution and 39-nt insertion were observed in single clones (Fig. 7). Interestingly, all GmPDS18g fragments from plant #5–1 shared the same 18-nt deletion upstream of and within PAM. And all GmPDS11g clones from plant #5–4 shared the same 25-nt deletion encompassing PAM (Fig. 7, Additional file 5: Table S3). It is worth noting that these deletions were observed in T0 plant #5 (Fig. 4), suggesting that they were inherited from the parental plant. Although the presence of transgene was not detected by PCR in plant #5–5, 31% of sequenced GmPDS18g clones and 10% of GmPDS11g clones showed sequence change. Similarly, in plant #5–6, mutations were detected in 40% of GmPDS18g clones and all of GmPDS11g clones (Table 3, Additional file 5: Table S3). These two plants demonstrate that mutations induced by CRISPR/Cas9 can be retained in progenies in which the transgene has been segregated. The same 18-nt deletion occurred in all mutant clones of plant #5–5, as well as mutant GmPDS18g clones of plant #5–6. All GmPDS11g clones of plant #5–6 also had the same 25-nt deletion in the target region (Fig. 7, Additional file 5: Table S3). Again, these deletions were observed in the parental plant (Fig. 4). No sequence change was detected in plant #5–3 at all, nor was the transgene, suggesting that it had reverted back to wild type through gene segregation.

**Table 3:** Summary of phenotypes and CRISPR/Cas9-induced mutations in GmPDS8 T1 line #5 plants

Plant	Phenotypes	Transgene detection	Indel frequency in GmPDS18g (%)	Indel frequency in GmPDS11g (%)
#5–1	Dwarf with albino leaves	+	100	93
#5–2	Dwarf with albino leaves	+	100	100
#5–3	Non-dwarf, with green leaves	−	0	0
#5–4	Dwarf with albino leaves	+	100	100
#5–5	Non-dwarf, with green leaves	−	31	10
#5–6	Non-dwarf, with green leaves	−	40	100

Indel frequency (%) is calculated as number of clones showing mutations divided by total number of sequenced clones

All T1 line #13 and #15 seeds germinated and developed into plants showing wild type phenotypes. The transgene was not detected in these plants. Targeted fragments from plant #13–1, 13–2, 13–3, 15–1, 15–2, 15–3, and 15–4 were sequenced, and no mutation was detected (Additional file 5: Table S3).

## Discussion

In recent years, CRISPR/Cas9-mediated genome editing has been employed in soybean to knock down genes involved in various traits, such as fatty acid and storage protein synthesis, plant height, node, stem, and flowering development, as well as altering ALS1 gene for herbicide resistance [27, 30–34, 36, 38]. Plants with altered traits were obtained, demonstrating the potential of using this technology for crop trait improvement. Nevertheless, utilization of genome editing in soybean is still not widespread, largely due to difficulties with genetic transformation or low mutation efficiencies.

Several groups have successfully mutagenized the PDS gene in Arabidopsis, rice, apple, tomato, grape, melon, cassava, etc., using CRISPR/Cas9 [13, 15, 18, 19, 41–43]. Similar work was conducted in soybean by Du et al. [21] using cv. Jack. The authors targeted each PDS gene specifically as well as both simultaneously. Different from our research, most of their constructs were assessed in hairy roots. One construct (D7) targeting both PDS genes was selected for stable transformation using cotyledonary nodes derived from 5-day-old seedlings as explants. Adventitious buds were regenerated, of which 5 out of 16 buds had dwarf and albino phenotypes, but the rest turned out to be false positives. Fully regenerated plants were not recovered in that study. We used ‘half-seed’ explants dissected from overnight-imbibed seeds for transformation of cv. Williams 82, based on the improved methodology described by Paz et al. [49]. T0 plants harboring mutations at desired loci were regenerated for all five constructs. Our genetic transformation system was reliable, with low occurrence of escapes overall. Transformation efficiency was as high as 7%. Very recently, Zhang et al. [37] also used similar transformation procedures for cv. Williams 82, and obtained a large number of transgenic plants. Both Du et al. [21] and Zhang et al. [37] used one guide RNA to induce mutations in the two PDS genes, similar to our GmPDS8 and GmPDS9 constructs. However, neither group evaluated targeting specificity as well as transmission of gene modifications beyond the T0 generation. Using simple constructs, we demonstrated efficient and specific editing of PDS genes in stably transformed soybean plants. Mutations and the associated phenotypes were transmitted to T1 progenies. Our simple and reliable system can be used as a reference to modify other genes for soybean trait improvement, contributing to the advancement of CRISPR/Cas9-mediated genome editing in soybean.

We obtained 75% to 100% mutation efficiencies for the five constructs. A few transgenic plants did not show mutations at target loci, likely because Cas9-induced DSB failed to take place, or the cleavage was correctly repaired by cellular DNA repair mechanisms. Observed mutations typically occurred a few nucleotides upstream of PAM, consistent with the location of Cas9-induced DSB [7, 9]. The vast majority were small deletions, although 1-nt insertion was also common. These indel types corroborate with the findings reported in earlier publications [33, 34, 36, 37]. As the guide sequences fall within the coding regions, such mutations caused missing amino acids, or frame shift, leading to altered amino acid sequences or premature termination of peptide synthesis. Indel frequencies varied among individual plants, with higher frequencies in plants exhibiting mutant phenotypes, consistent with the findings reported by Zhang et al. [37]. In a few severely dwarf and albino GmPDS8 and GmPDS9 T0 plantlets, 100% indel frequencies in both PDS genes were obtained. Whereas in GmPDS8 and GmPDS9 plants without visible mutant phenotypes, indel frequencies were low. These plants could be chimeras. Previous studies compared the targeting efficiencies using the soybean U6 promoter vs. Arabidopsis U6 promoter, and reported increased efficiency using the former [21, 23]. Evaluation of several soybean U6 promoters for mutation efficiencies was also undertaken [25]. Switching to an efficient soybean U6 promoter for sgRNA expression could further improve the CRISPR/Cas9 system.

One of the concerns with CRISPR/Cas9 is off-target effect [50]. Although we did not perform full genome sequencing for full off-target analysis, our assessments suggested specific targeting of PDS genes. First, we sequenced both GmPDS11g and GmPDS18g fragments in examined plants. GmPDS1 and GmPDS3 target GmPDS18g specifically, each guide RNA contains 3 mismatches to the GmPDS11g counterpart. In both cases, the 3 interspaced mismatches occur at positions within 14-nt at the 3’ end of the 20-nt guide sequence. Mutation was not detected in the sequenced GmPDS11g fragments. Similarly, GmPDS7 targets GmPDS11g specifically, also with 3 interspaced mismatches to the GmPDS18g counterpart. One mismatch is 18-nt away, and two mismatches within 14-nt upstream of PAM. No mutation was detected in the GmPDS18g counterpart either. As expected, plants transformed with the constructs targeting one PDS gene specifically were phenotypically similar to wild type, as disruption in one paralogue could be functionally compensated by the other. For additional analysis, we examined several potential off-target loci outside of PDS genes, with sequence similarities to GmPDS8 guide RNA, followed by PAM ‘-NGG’. ARF10 has 5 consecutive mismatches located immediately upstream of PAM. Its homologue ARF13 contains 10 consecutive mismatches, also at the 3’ end. GGT10 and GGT20 have 7 and 6 interspaced mismatches, respectively; both contain 4 consecutive mismatches directly upstream of PAM. No mutation was detected at these sites among all sequenced clones, providing further evidence for gene editing specificity. It has been revealed that single base specificity ranges from 8–14 bp directly upstream of PAM (named PAM proximal region), whereas mismatches at 5’ end of the guide sequence are more tolerated [12, 51]. Five consecutive mismatches or at least three interspaced mismatches eliminated detectable off-targeting in most cases [51]. To maximize specificity, guide sequences are recommended to contain a maximal number of consecutive mismatches to the off-target sites, or at least three mismatches spaced less than four bases apart, among which at least two should be located within the PAM-proximal region [51]. Our guide sequences selected by CRISPR-PLANT online tool [45] satisfy most of the above conditions, thus enabling specific targeting. It has also been shown that high concentration of Cas9/sgRNA complex results higher off-target incidences [51, 52]. The use of tissue specific or inducible promoter to drive the expression of Cas9 can be used to further minimize off-target effect.

We examined T1 plants derived from three independently transformed GmPDS8 lines for inheritance of mutant phenotypes as well as gene modifications at target loci. Three plants of line #5 exhibited dwarf and albino phenotypes. Transgene was detected in each, and indel frequencies were 100% in GmPDS18g, as well as 93% and 100% in GmPDS11g. Two other plants of the same line did not show visible mutant phenotypes. PCR analysis showed that the transgene was not present. Interestingly, the 18-nt and 25-nt deletions present in the T0 parent were detected in these plants uniformly among GmPDS18g or GmPDS11g fragments, indicating that mutations induced by CRISPR/Cas9 were transmitted to progenies while the transgene was segregated out. This inheritance pattern is the most desired for crop trait improvement as the plants are transgene free. However, in the case of line #13 and #15, both the transgene and mutations were not inherited in the T1 progenies. Indel frequencies in the T0 generation were low in these two lines, thus it is likely that the T0 plants were chimeric and the mutations were only present in somatic cells, thus not passed down to the T1 generation. Michno et al. [35] examined integration and inheritance patterns in CRISPR/Cas9 soybean lines, and reported several patterns including transmission of mutations but segregation of transgenes, no transmission of transgenes and mutations, and inheritance of transgenes located within the target sites. The loss of transgenes and mutations in subsequent generations appear to be common for CRISPR/Cas9-induced mutagenesis [35]. Methods to increase mutation transmission are being explored, such as the use of germ-cell specific promoters to drive the expression of Cas9 [29].

## Conclusions

We demonstrated a simple, efficient, and specific genome editing system by targeting PDS genes with CRISPR/Cas9 in stably transformed soybean plants. Simultaneous targeting of both PDS genes using one guide RNA led to the development of dwarf and albino phenotypes. Induced gene modifications were transmitted to the T1 generation, even in progenies that lost the transgene through segregation. Our CRISPR/Cas9-mediated genome editing system can be employed to modify other genes for soybean trait improvement.

## Methods

### Plasmid construction

Nucleotide and peptide sequences of the two homologous soybean PDS genes were obtained from Phytozome v.12.1 [46]. Guide sequences targeting each gene specifically were selected using CRISPR-PLANT online tool [45]. Sequences targeting both PDS genes simultaneously were designed manually by selecting 20 nucleotides on exons in the upstream locations of the genes, in the conserved regions preceding PAM ‘-NGG-’. Selected nucleotide sequences were then used as query in BLAST against the soybean genome in Phytozome to check for specificity. Each 20-nt guide sequence was included as part of the forward primer used to amplify a sgRNA-U6 terminator (TAtU6) fragment (Additional file 7: Table S5), using an existing sgRNA-TAtU6 plasmid as template [48]. The fragment was amplified by PCR using Phusion high fidelity DNA polymerase (New England Biolabs). The PCR reaction consisted of the following steps: initial denaturation at 98 °C for 30 s (sec), 32 cycles of 98 °C for 10 s, 58 °C for 30 s, and 72 °C for 30 s, followed by a final extension at 72 °C for 7 min (min). A-tail was then added to the fragment using GoTaq Flexi taq DNA polymerase (Promega) at 70 °C for 30 min, allowing it to be ligated into T-vector using the Promega pGEM-T Easy Vector system (Promega). A 35S Cauliflower Mosaic Virus (CaMV) promoter, maize codon-optimized Cas9, eGFP, Nos terminator, Arabidopsis AtU6 promoter, and sgRNA-U6 terminator were assembled into Gateway-compatible entry vector pGateG according to pre-determined order using Goldengate ligation system [47, 48]. Individual ‘modules’ with specific overhangs containing various components of the Cas9/sgRNA assembly were generated previously [48]. The ligation reaction consisted of 0.5μL of each ‘module’ and pGateG vector at approximately 150 ng/μL concentration, 0.5μL 10X T4 ligase buffer, 0.5μL 10X BSA, 0.3μL BsaI-HF (New England Biolabs), and 0.3μL T4 DNA ligase (New England Biolabs). The reaction was run in a thermocycler using 50 cycles of 37 °C for 5 min, and 16 °C for 5 min, followed by one step of 50 °C for 5 min, and 80 °C for 10 min. The resulting Cas9/sgRNA expression cassette was transferred into binary vector pEarleygate301 (pEG301) [53] using Gateway LR Clonase II Enzyme mix (Invitrogen) according to manufacturer’s instructions.

### Genetic transformation of soybean

Genome editing constructs were transferred into *Agrobacterium tumefaciens* strain EHA105 using electroporation. pEG301 contains aminoglycoside phosphotransferase gene that confers resistance to kanamycin in bacteria, as well as phosphinothricin acetyltransferase gene for BASTA selection in plants. A few *A. tumefaciens* colonies on Luria–Bertani (LB) (Difco) medium containing 50 mg/L kanamycin and 25 mg/L rifampicin (Sigma-Aldrich) were randomly selected for verification by PCR using vector specific primers (Additional file 7: Table S5). Colonies that showed positive PCR results were inoculated into 5 mL LB broth as start-up culture with the same antibiotic selections, grown overnight with shaking at 28 °C, 180 rpm. On the following day, 30-50μL of the start-up culture was transferred into 200 mL fresh Yeast Extract Beef broth (YEB) (PhytoTechnology) containing 50 mg/L kanamycin, 25 mg/L rifampicin, and 100 μM acetosyringone (Sigma-Aldrich), grown overnight with shaking at 28 °C, 180 rpm, until the O.D._600_ reached 0.8–1.0. On the day of transformation, bacteria pellet was collected by centrifugation at 5000 rpm for 10 min at 4 °C, washed once with liquid co-cultivation (LCC) medium (1/10 Gamborg B-5 basal salts, B5 vitamins, 20 mM 2-(N-morpholino)ethanesulfonic acid (MES), 3% sucrose, 7.5 μM 6-benzylaminopurine (BAP), 0.7 μM gibberellic acid (GA), 200 μM acetosyringone, pH 5.4) (Bioshop, Phytotechnology, Sigma-Aldrich), and resuspended in half of the original volume in LCC medium, for a final O.D._600_ of 1.5.

Soybean cv. Williams 82 seeds were kindly provided by Dr. Aiming Wang at Agriculture and Agri-Food Canada. Seeds were surface sterilized overnight in a desiccator using chlorine gas produced by mixing 5 mL of 12 N hydrochloric acid (HCl) (Sigma-Aldrich) with commercial bleach containing 6% (w/v) sodium hypochlorite. Transformation procedures were adapted from Paz et al. [49] and Olhoft et al. [54]. Disinfected seeds were soaked overnight in sterile ddH_2_O, at room temperature in dark. On the day of transformation, seed coat was removed, and each embryo was cut longitudinally along the hilum, resulting separate cotyledons attached to the halved embryo axis. Small axillary shoots, if present, were removed, and the node at the junction of cotyledon and embryo axis was gently wounded with a scalpel. In each 100 × 20 mm petri dish, about 60 explants were immersed for 30 min in infection broth containing *Agrobacterium* carrying the constructs, at room temperature with gentle shaking at 50 rpm. Afterwards, explants were blotted dry on sterile filter paper, and placed flat side down on co-cultivation medium (1/10 Gamborg B-5 basal salts, B5 vitamins, 20 mM MES, 3% sucrose, 7.4 μM BAP, 0.7 μM GA, 400 mg/L L-cysteine, 154 mg/L dithiothreitol (DTT), 158 mg/L sodium-thiosulfate, 200 μM acetosyringone, pH 5.4 with 0.7% agar) for 5 days. Tissue culture was carried out at 24 °C under 16-h photoperiod with 100μmoles/s/m^2^ illumination. Following co-cultivation, explants were washed 2 times in wash medium (Gamborg B-5 basal medium, 3% sucrose, 3 mM MES, 7.4 μM BAP, 1.4 μM GA, 50 mg/L cefotaxime, 300 mg/L timentin, pH 5.6), and transferred to shoot inducing medium (SIM) (Gamborg B-5 basal medium, 3% sucrose, 3 mM MES, 7.4 μM BAP, 50 mg/L cefotaxime, 300 mg/L timentin, pH 5.6, with 0.7% agar) initially without selection for two weeks, placed flat side up with the base section embedded in the medium. Subsequently, explants were transferred to SIM containing 6-10 mg/L herbicide glufosinate-ammonium, also known as BASTA, for selection. Surviving shoot buds along with the base were removed from the original explants, and cultured on shoot elongation medium (SEM) (MS basal salts, B5 vitamins, 3% sucrose, 3 mM MES, 2.8 μM zeatin riboside, 1.4 μM GA, 0.6 μM indole-3-acetic acid (IAA), 50 mg/L L-asparagine monohydrate, 100 mg/L L-pyroglutamic acid, 75 mg/L cefotaxime, 300 mg/L timentin, 5 mg/L BASTA, pH 5.6, with 0.7% agar). Elongated shoots (> 2 cm) were excised from shoot pads, dipped in sterile 0.5–1 mg/mL indole-3-butyric acid (IBA) and transferred to the rooting medium (RM) (MS basal salts, B5 vitamins, 2% sucrose, 3 mM MES, 50 mg/L L-asparagine monohydrate, 100 mg/L L-pyroglutamic acid, 75 mg/L cefotaxime, 300 mg/L timentin, pH5.6, with 0.3% Phytagel). Rooted plantlets were separated from agar, rinsed with water, and transplanted to PRO-MIX BX soil, grown in growth cabinets at 24 °C under 16-h photoperiod with 200μmoles/s/m^2^ illumination.

### Molecular characterization

To extract total genomic DNA, leaf tissues from plants growing in soil or from regenerating shoots were collected and homogenized using Qiagen Tissue Lyser II homogenizer. Homogenized tissue was resuspended in DNA extraction buffer made of 2% hexadecyltrimethylammonium bromide (CTAB), 100 mM Tris pH 8.0, 20 mM ethylenediaminetetraacetic acid (EDTA) pH 8.0, 1.4 M sodium chloride (NaCl), 2% polyvinylpyrrolidone (PVP)-40 (Sigma-Aldrich), followed by centrifugation at 5000 rpm for 10 min to settle the debris. The supernatant was incubated at 65 °C for 1 h before mixed with 1 volume of chloroform:isoamyl alcohol (24:1) (Sigma-Aldrich), then centrifuged at 13000 rpm for 10 min to separate the aqueous and organic phases. The aqueous phase was mixed with 1 volume of isopropanol (Sigma-Aldrich) for DNA precipitation. Following centrifugation at 13000 rpm for 10 min at 4 °C, DNA pellet was washed with 70% ethanol. The final DNA pellet was dried in a 60 °C oven for 10 min, and resuspended in sterile ddH_2_O. To verify transformation, genomic DNA was used as template for PCR using Promega GoTaq Flexi taq DNA polymerase with construct-specific primers (Additional file 7: Table S5). The PCR reaction consisted of the following steps: initial denaturation at 95 °C for 5 min, 35 cycles of 95 °C for 45 s, 58 °C for 45 s, and 72 °C for 1 min, followed by a final extension at 72 °C for 7 min. To detect mutations, target gene fragments were amplified by PCR using Phusion high fidelity DNA polymerase (New England Biolabs) under the conditions described earlier, and cloned into pGateG vector through Golden Gate assembly. Primers used to amplify the target fragments are included in Additional file 7: Table S5. Fragments assembled in pGateG were transferred into *E.coli* strain DH5ɑ by electroporation. Plasmids were extracted from randomly selected *E.coli* colonies using QIAprep Spin Miniprep Kit (Qiagen) according to manufacturer’s procedures. Extracted plasmids were sent to Eurofins Genomics for Sanger sequencing.

### Off-target analysis outside of PDS genes

The 20-nt GmPDS8 guide sequence was used as query for nucleotide BLAST provided by National Center for Biotechnology Information (NCBI) [55, 56] to search for similar sequences in the soybean genome. The non-PDS loci with top alignment scores were identified as potential off-targets. Genomic sequences of the associated genes were obtained from Phytozome. To evaluate off-target occurrence, gene fragments containing the potential off-target sites were amplified from genomic DNA of GmPDS8 T0 plant #5 and #6C, assembled into plasmid pGateG, and sequenced from randomly selected *E.coli* clones, according to procedures described above.

### Sequence alignment

Nucleotide and peptide sequences of GmPDS11g and GmPDS18g coding regions were aligned using MegAlign Pro 17 software, using ClustalW method.

## Supplementary Information

**Additional file 1.** **Figure S1**. Alignment of GmPDS11g and GmPDS18g nucleotide coding sequences.

**Additional file 2.** **Figure S2**. Alignment of GmPDS11g and GmPDS18g peptide sequences.

**Additional file 3.** **Table S1**. Summary of soybean genetic transformations with genome editing constructs.

**Additional file 4.** **Table S2**. Detection of mutations in T0 plants.

**Additional file 5.** **Table S3**. Detection of mutations in GmPDS8 T1 plants.

**Additional file 6.** **Table S4**. Off-target analysis in GmPDS8 T0 plants.

**Additional file 7.** **Table S5**. Primers used for cloning and molecular analyses.

**Additional file 8.** **Figure S3**. Example agarose gels showing detection of transgene in GmPDS8 T1 plants.

## Data Availability

The datasets used and/or analysed during the current study are available from the corresponding author on reasonable request.

## Abbreviations

BAP: 6-Benzylaminopurine
BLAST: Basic Local Alignment Search Tool
bp: Base pair
Cas9: CRISPR-associated 9
CRISPR: Clustered regularly interspaced short palindromic repeats
crRNA: CRISPR RNA
cv: Cultivar
DSB: Double stranded break
GA: Gibberellic acid
HDR: Homology-directed repair
IAA: Indole-3-acetic acid
IBA: Indole-3-butyric acid
MES: 2-(N-morpholino)ethanesulfonic acid
min: Minutes
NHEJ: Nonhomologous end joining
nt: Nucleotide
PAM: Protospacer adjacent motif
PCR: Polymerase chain reaction
PDS: Phytoene desaturase
pEG301: PEarleygate 301
sec: Seconds
sgRNA: Single guide RNA
tracrRNA: Trans-activating crRNA
