# Supplementary material for: An efficient and specific CRISPR-Cas9 genome editing system targeting soybean phytoene desaturase genes
Source: BMC Biotechnol. 2022 Feb 15;22:7. doi: 10.1186/s12896-022-00737-7 (PMC8845245; doi:10.1186/s12896-022-00737-7)
Supplement: Supplementary file 1 — Additional file 1. Figure S1. Alignment of GmPDS11g and GmPDS18g nucleotide coding sequences. [file 12896_2022_737_MOESM1_ESM.pptx]

## Slide 1
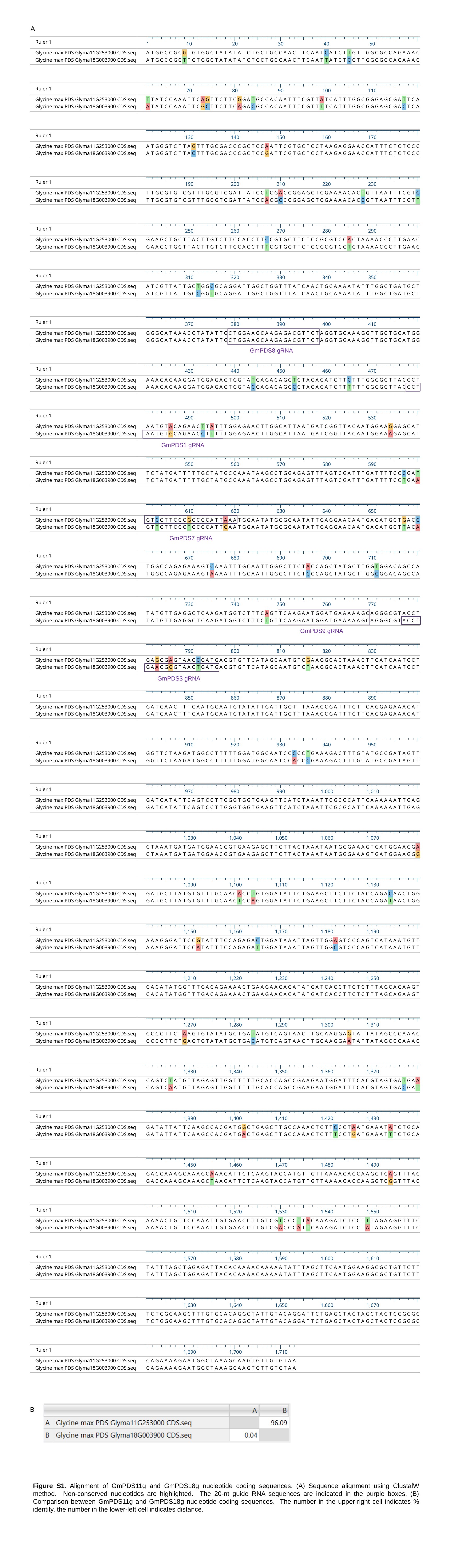

A
1
10
20
30
40
50
Ruler 1
A
T
G
G
C
C
G
C
G
T
G
T
G
G
C
T
A
T
A
T
A
T
C
T
G
C
T
G
C
C
A
A
C
T
T
C
A
A
T
C
A
T
C
T
T
G
T
T
G
G
C
G
C
C
A
G
A
A
A
C
Glycine max PDS Glyma11G253000 CDS.seq
A
T
G
G
C
C
G
C
T
T
G
T
G
G
C
T
A
T
A
T
A
T
C
T
G
C
T
G
C
C
A
A
C
T
T
C
A
A
T
T
A
T
C
T
C
G
T
T
G
G
C
G
C
C
A
G
A
A
A
C
Glycine max PDS Glyma18G003900 CDS.seq
70
80
90
100
110
Ruler 1
T
T
A
T
C
C
A
A
A
T
T
C
A
G
T
T
C
T
T
C
G
G
A
T
G
C
C
A
C
A
A
T
T
T
C
G
T
T
A
T
C
A
T
T
T
G
G
C
G
G
G
A
G
C
G
A
T
T
C
A
Glycine max PDS Glyma11G253000 CDS.seq
A
T
A
T
C
C
A
A
A
T
T
C
G
C
T
T
C
T
T
C
A
G
A
C
G
C
C
A
C
A
A
T
T
T
C
G
T
T
T
T
C
A
T
T
T
G
G
C
G
G
G
A
G
C
G
A
C
T
C
A
Glycine max PDS Glyma18G003900 CDS.seq
130
140
150
160
170
Ruler 1
A
T
G
G
G
T
C
T
T
A
G
T
T
T
G
C
G
A
C
C
C
G
C
T
C
C
A
A
T
T
C
G
T
G
C
T
C
C
T
A
A
G
A
G
G
A
A
C
C
A
T
T
T
C
T
C
T
C
C
C
Glycine max PDS Glyma11G253000 CDS.seq
A
T
G
G
G
T
C
T
T
A
C
T
T
T
G
C
G
A
C
C
C
G
C
T
C
C
G
A
T
T
C
G
T
G
C
T
C
C
T
A
A
G
A
G
G
A
A
C
C
A
T
T
T
C
T
C
T
C
C
C
Glycine max PDS Glyma18G003900 CDS.seq
190
200
210
220
230
Ruler 1
T
T
G
C
G
T
G
T
C
G
T
T
T
G
C
G
T
C
G
A
T
T
A
T
C
C
T
C
G
A
C
C
G
G
A
G
C
T
C
G
A
A
A
A
C
A
C
T
G
T
T
A
A
T
T
T
C
G
T
C
Glycine max PDS Glyma11G253000 CDS.seq
T
T
G
C
G
T
G
T
C
G
T
T
T
G
C
G
T
C
G
A
T
T
A
T
C
C
A
C
G
C
C
C
G
G
A
G
C
T
C
G
A
A
A
A
C
A
C
C
G
T
T
A
A
T
T
T
C
G
T
T
Glycine max PDS Glyma18G003900 CDS.seq
250
260
270
280
290
Ruler 1
G
A
A
G
C
T
G
C
T
T
A
C
T
T
G
T
C
T
T
C
C
A
C
C
T
T
C
C
G
T
G
C
T
T
C
T
C
C
G
C
G
T
C
C
A
C
T
A
A
A
A
C
C
C
T
T
G
A
A
C
Glycine max PDS Glyma11G253000 CDS.seq
G
A
A
G
C
T
G
C
T
T
A
C
T
T
G
T
C
T
T
C
C
A
C
C
T
T
T
C
G
T
G
C
T
T
C
T
C
C
G
C
G
T
C
C
T
C
T
A
A
A
A
C
C
C
T
T
G
A
A
C
Glycine max PDS Glyma18G003900 CDS.seq
310
320
330
340
350
Ruler 1
A
T
C
G
T
T
A
T
T
G
C
T
G
G
C
G
C
A
G
G
A
T
T
G
G
C
T
G
G
T
T
T
A
T
C
A
A
C
T
G
C
A
A
A
A
T
A
T
T
T
G
G
C
T
G
A
T
G
C
T
Glycine max PDS Glyma11G253000 CDS.seq
A
T
C
G
T
T
A
T
T
G
C
C
G
G
T
G
C
A
G
G
A
T
T
G
G
C
T
G
G
T
T
T
A
T
C
A
A
C
T
G
C
A
A
A
A
T
A
T
T
T
G
G
C
T
G
A
T
G
C
T
Glycine max PDS Glyma18G003900 CDS.seq
370
380
390
400
410
Ruler 1
G
G
G
C
A
T
A
A
A
C
C
T
A
T
A
T
T
G
C
T
G
G
A
A
G
C
A
A
G
A
G
A
C
G
T
T
C
T
A
G
G
T
G
G
A
A
A
G
G
T
T
G
C
T
G
C
A
T
G
G
Glycine max PDS Glyma11G253000 CDS.seq
G
G
G
C
A
T
A
A
A
C
C
T
A
T
A
T
T
G
C
T
G
G
A
A
G
C
A
A
G
A
G
A
C
G
T
T
C
T
A
G
G
T
G
G
A
A
A
G
G
T
T
G
C
T
G
C
A
T
G
G
Glycine max PDS Glyma18G003900 CDS.seq
430
440
450
460
470
Ruler 1
A
A
A
G
A
C
A
A
G
G
A
T
G
G
A
G
A
C
T
G
G
T
A
T
G
A
G
A
C
A
G
G
T
C
T
A
C
A
C
A
T
C
T
T
C
T
T
T
G
G
G
G
C
T
T
A
C
C
C
T
Glycine max PDS Glyma11G253000 CDS.seq
A
A
A
G
A
C
A
A
G
G
A
T
G
G
A
G
A
C
T
G
G
T
A
C
G
A
G
A
C
A
G
G
C
C
T
A
C
A
C
A
T
C
T
T
T
T
T
T
G
G
G
G
C
T
T
A
C
C
C
T
Glycine max PDS Glyma18G003900 CDS.seq
490
500
510
520
530
Ruler 1
A
A
T
G
T
A
C
A
G
A
A
C
T
T
A
T
T
T
G
G
A
G
A
A
C
T
T
G
G
C
A
T
T
A
A
T
G
A
T
C
G
G
T
T
A
C
A
A
T
G
G
A
A
G
G
A
G
C
A
T
Glycine max PDS Glyma11G253000 CDS.seq
A
A
T
G
T
G
C
A
G
A
A
C
C
T
T
T
T
T
G
G
A
G
A
A
C
T
T
G
G
C
A
T
T
A
A
T
G
A
T
C
G
G
T
T
A
C
A
A
T
G
G
A
A
A
G
A
G
C
A
T
Glycine max PDS Glyma18G003900 CDS.seq
550
560
570
580
590
Ruler 1
T
C
T
A
T
G
A
T
T
T
T
T
G
C
T
A
T
G
C
C
A
A
A
T
A
A
G
C
C
T
G
G
A
G
A
G
T
T
T
A
G
T
C
G
A
T
T
T
G
A
T
T
T
T
C
C
C
G
A
T
Glycine max PDS Glyma11G253000 CDS.seq
T
C
T
A
T
G
A
T
T
T
T
T
G
C
T
A
T
G
C
C
A
A
A
T
A
A
G
C
C
T
G
G
A
G
A
G
T
T
T
A
G
T
C
G
A
T
T
T
G
A
T
T
T
T
C
C
T
G
A
A
Glycine max PDS Glyma18G003900 CDS.seq
610
620
630
640
650
Ruler 1
G
T
C
C
T
T
C
C
C
G
C
C
C
C
A
T
T
A
A
A
T
G
G
A
A
T
A
T
G
G
G
C
A
A
T
A
T
T
G
A
G
G
A
A
C
A
A
T
G
A
G
A
T
G
C
T
G
A
C
C
Glycine max PDS Glyma11G253000 CDS.seq
G
T
T
C
T
T
C
C
C
T
C
C
C
C
A
T
T
G
A
A
T
G
G
A
A
T
A
T
G
G
G
C
A
A
T
A
T
T
G
A
G
G
A
A
C
A
A
T
G
A
G
A
T
G
C
T
T
A
C
A
Glycine max PDS Glyma18G003900 CDS.seq
670
680
690
700
710
Ruler 1
T
G
G
C
C
A
G
A
G
A
A
A
G
T
C
A
A
A
T
T
T
G
C
A
A
T
T
G
G
G
C
T
T
C
T
A
C
C
A
G
C
T
A
T
G
C
T
T
G
G
T
G
G
A
C
A
G
C
C
A
Glycine max PDS Glyma11G253000 CDS.seq
T
G
G
C
C
A
G
A
G
A
A
A
G
T
A
A
A
A
T
T
T
G
C
A
A
T
T
G
G
G
C
T
T
C
T
C
C
C
A
G
C
T
A
T
G
C
T
T
G
G
C
G
G
A
C
A
G
C
C
A
Glycine max PDS Glyma18G003900 CDS.seq
730
740
750
760
770
Ruler 1
T
A
T
G
T
T
G
A
G
G
C
T
C
A
A
G
A
T
G
G
T
C
T
T
T
C
A
G
T
T
C
A
A
G
A
A
T
G
G
A
T
G
A
A
A
A
A
G
C
A
G
G
G
C
G
T
A
C
C
T
Glycine max PDS Glyma11G253000 CDS.seq
T
A
T
G
T
T
G
A
G
G
C
T
C
A
A
G
A
T
G
G
T
C
T
T
T
C
T
G
T
T
C
A
A
G
A
A
T
G
G
A
T
G
A
A
A
A
A
G
C
A
G
G
G
C
G
T
A
C
C
T
Glycine max PDS Glyma18G003900 CDS.seq
790
800
810
820
830
Ruler 1
G
A
G
C
G
A
G
T
A
A
C
C
G
A
T
G
A
G
G
T
G
T
T
C
A
T
A
G
C
A
A
T
G
T
C
G
A
A
G
G
C
A
C
T
A
A
A
C
T
T
C
A
T
C
A
A
T
C
C
T
Glycine max PDS Glyma11G253000 CDS.seq
G
A
A
C
G
G
G
T
A
A
C
T
G
A
T
G
A
G
G
T
G
T
T
C
A
T
A
G
C
A
A
T
G
T
C
T
A
A
G
G
C
A
C
T
A
A
A
C
T
T
C
A
T
C
A
A
T
C
C
T
Glycine max PDS Glyma18G003900 CDS.seq
850
860
870
880
890
Ruler 1
G
A
T
G
A
A
C
T
T
T
C
A
A
T
G
C
A
A
T
G
T
A
T
A
T
T
G
A
T
T
G
C
T
T
T
A
A
A
C
C
G
A
T
T
T
C
T
T
C
A
G
G
A
G
A
A
A
C
A
T
Glycine max PDS Glyma11G253000 CDS.seq
G
A
T
G
A
A
C
T
T
T
C
A
A
T
G
C
A
A
T
G
T
A
T
A
T
T
G
A
T
T
G
C
T
T
T
A
A
A
C
C
G
A
T
T
T
C
T
T
C
A
G
G
A
G
A
A
A
C
A
T
Glycine max PDS Glyma18G003900 CDS.seq
910
920
930
940
950
Ruler 1
G
G
T
T
C
T
A
A
G
A
T
G
G
C
C
T
T
T
T
T
G
G
A
T
G
G
C
A
A
T
C
C
C
C
C
T
G
A
A
A
G
A
C
T
T
T
G
T
A
T
G
C
C
G
A
T
A
G
T
T
Glycine max PDS Glyma11G253000 CDS.seq
G
G
T
T
C
T
A
A
G
A
T
G
G
C
C
T
T
T
T
T
G
G
A
T
G
G
C
A
A
T
C
C
A
C
C
C
G
A
A
A
G
A
C
T
T
T
G
T
A
T
G
C
C
G
A
T
A
G
T
T
Glycine max PDS Glyma18G003900 CDS.seq
970
980
990
1,000
1,010
Ruler 1
G
A
T
C
A
T
A
T
T
C
A
G
T
C
C
T
T
G
G
G
T
G
G
T
G
A
A
G
T
T
C
A
T
C
T
A
A
A
T
T
C
G
C
G
C
A
T
T
C
A
A
A
A
A
A
T
T
G
A
G
Glycine max PDS Glyma11G253000 CDS.seq
G
A
T
C
A
T
A
T
T
C
A
G
T
C
C
T
T
G
G
G
T
G
G
T
G
A
A
G
T
T
C
A
T
C
T
A
A
A
T
T
C
G
C
G
C
A
T
T
C
A
A
A
A
A
A
T
T
G
A
G
Glycine max PDS Glyma18G003900 CDS.seq
1,030
1,040
1,050
1,060
1,070
Ruler 1
C
T
A
A
A
T
G
A
T
G
A
T
G
G
A
A
C
G
G
T
G
A
A
G
A
G
C
T
T
C
T
T
A
C
T
A
A
A
T
A
A
T
G
G
G
A
A
A
G
T
G
A
T
G
G
A
A
G
G
A
Glycine max PDS Glyma11G253000 CDS.seq
C
T
A
A
A
T
G
A
T
G
A
T
G
G
A
A
C
G
G
T
G
A
A
G
A
G
C
T
T
C
T
T
A
C
T
A
A
A
T
A
A
T
G
G
G
A
A
A
G
T
G
A
T
G
G
A
A
G
G
G
Glycine max PDS Glyma18G003900 CDS.seq
1,090
1,100
1,110
1,120
1,130
Ruler 1
G
A
T
G
C
T
T
A
T
G
T
G
T
T
T
G
C
A
A
C
A
C
C
T
G
T
G
G
A
T
A
T
T
C
T
G
A
A
G
C
T
T
C
T
T
C
T
A
C
C
A
G
A
C
A
A
C
T
G
G
Glycine max PDS Glyma11G253000 CDS.seq
G
A
T
G
C
T
T
A
T
G
T
G
T
T
T
G
C
A
A
C
T
C
C
A
G
T
G
G
A
T
A
T
T
C
T
G
A
A
G
C
T
T
C
T
T
C
T
A
C
C
A
G
A
T
A
A
C
T
G
G
Glycine max PDS Glyma18G003900 CDS.seq
1,150
1,160
1,170
1,180
1,190
Ruler 1
A
A
A
G
G
G
A
T
T
C
C
G
T
A
T
T
T
C
C
A
G
A
G
A
C
T
G
G
A
T
A
A
A
T
T
A
G
T
T
G
G
A
G
T
C
C
C
A
G
T
C
A
T
A
A
A
T
G
T
T
Glycine max PDS Glyma11G253000 CDS.seq
A
A
A
G
G
G
A
T
T
C
C
A
T
A
T
T
T
C
C
A
G
A
G
A
T
T
G
G
A
T
A
A
A
T
T
A
G
T
T
G
G
C
G
T
C
C
C
A
G
T
C
A
T
A
A
A
T
G
T
T
Glycine max PDS Glyma18G003900 CDS.seq
1,210
1,220
1,230
1,240
1,250
Ruler 1
C
A
C
A
T
A
T
G
G
T
T
T
G
A
C
A
G
A
A
A
A
C
T
G
A
A
G
A
A
C
A
C
A
T
A
T
G
A
T
C
A
C
C
T
T
C
T
C
T
T
T
A
G
C
A
G
A
A
G
T
Glycine max PDS Glyma11G253000 CDS.seq
C
A
C
A
T
A
T
G
G
T
T
T
G
A
C
A
G
A
A
A
A
C
T
G
A
A
G
A
A
C
A
C
A
T
A
T
G
A
T
C
A
C
C
T
T
C
T
C
T
T
T
A
G
C
A
G
A
A
G
T
Glycine max PDS Glyma18G003900 CDS.seq
1,270
1,280
1,290
1,300
1,310
Ruler 1
C
C
C
C
T
T
C
T
A
A
G
T
G
T
A
T
A
T
G
C
T
G
A
T
A
T
G
T
C
A
G
T
A
A
C
T
T
G
C
A
A
G
G
A
G
T
A
T
T
A
T
A
G
C
C
C
A
A
A
C
Glycine max PDS Glyma11G253000 CDS.seq
C
C
C
C
T
T
C
T
G
A
G
T
G
T
A
T
A
T
G
C
T
G
A
C
A
T
G
T
C
A
G
T
A
A
C
T
T
G
C
A
A
G
G
A
A
T
A
T
T
A
T
A
G
C
C
C
A
A
A
C
Glycine max PDS Glyma18G003900 CDS.seq
1,330
1,340
1,350
1,360
1,370
Ruler 1
C
A
G
T
C
T
A
T
G
T
T
A
G
A
G
T
T
G
G
T
T
T
T
T
G
C
A
C
C
A
G
C
C
G
A
A
G
A
A
T
G
G
A
T
T
T
C
A
C
G
T
A
G
T
G
A
T
G
A
A
Glycine max PDS Glyma11G253000 CDS.seq
C
A
G
T
C
A
A
T
G
T
T
A
G
A
G
T
T
G
G
T
T
T
T
T
G
C
A
C
C
A
G
C
C
G
A
A
G
A
A
T
G
G
A
T
T
T
C
A
C
G
T
A
G
T
G
A
C
G
A
T
Glycine max PDS Glyma18G003900 CDS.seq
1,390
1,400
1,410
1,420
1,430
Ruler 1
G
A
T
A
T
T
A
T
T
C
A
A
G
C
C
A
C
G
A
T
G
G
C
T
G
A
G
C
T
T
G
C
C
A
A
A
C
T
C
T
T
C
C
C
T
A
A
T
G
A
A
A
T
A
T
C
T
G
C
A
Glycine max PDS Glyma11G253000 CDS.seq
G
A
T
A
T
T
A
T
T
C
A
A
G
C
C
A
C
G
A
T
G
A
C
T
G
A
G
C
T
T
G
C
C
A
A
A
C
T
C
T
T
T
C
C
T
G
A
T
G
A
A
A
T
T
T
C
T
G
C
A
Glycine max PDS Glyma18G003900 CDS.seq
1,450
1,460
1,470
1,480
1,490
Ruler 1
G
A
C
C
A
A
A
G
C
A
A
A
G
C
A
A
A
G
A
T
T
C
T
C
A
A
G
T
A
C
C
A
T
G
T
T
G
T
T
A
A
A
A
C
A
C
C
A
A
G
G
T
C
A
G
T
T
T
A
C
Glycine max PDS Glyma11G253000 CDS.seq
G
A
C
C
A
A
A
G
C
A
A
A
G
C
T
A
A
G
A
T
T
C
T
C
A
A
G
T
A
C
C
A
T
G
T
T
G
T
T
A
A
A
A
C
A
C
C
A
A
G
G
T
C
G
G
T
T
T
A
C
Glycine max PDS Glyma18G003900 CDS.seq
1,510
1,520
1,530
1,540
1,550
Ruler 1
A
A
A
A
C
T
G
T
T
C
C
A
A
A
T
T
G
T
G
A
A
C
C
T
T
G
T
C
G
T
C
C
C
T
T
A
C
A
A
A
G
A
T
C
T
C
C
T
T
T
A
G
A
A
G
G
T
T
T
C
Glycine max PDS Glyma11G253000 CDS.seq
A
A
A
A
C
T
G
T
T
C
C
A
A
A
T
T
G
T
G
A
A
C
C
T
T
G
T
C
G
A
C
C
C
A
T
T
C
A
A
A
G
A
T
C
T
C
C
T
A
T
A
G
A
A
G
G
T
T
T
C
Glycine max PDS Glyma18G003900 CDS.seq
1,570
1,580
1,590
1,600
1,610
Ruler 1
T
A
T
T
T
A
G
C
T
G
G
A
G
A
T
T
A
C
A
C
A
A
A
A
C
A
A
A
A
A
T
A
T
T
T
A
G
C
T
T
C
A
A
T
G
G
A
A
G
G
C
G
C
T
G
T
T
C
T
T
Glycine max PDS Glyma11G253000 CDS.seq
T
A
T
T
T
A
G
C
T
G
G
A
G
A
T
T
A
C
A
C
A
A
A
A
C
A
A
A
A
A
T
A
T
T
T
A
G
C
T
T
C
A
A
T
G
G
A
A
G
G
C
G
C
T
G
T
T
C
T
T
Glycine max PDS Glyma18G003900 CDS.seq
1,630
1,640
1,650
1,660
1,670
Ruler 1
T
C
T
G
G
G
A
A
G
C
T
T
T
G
T
G
C
A
C
A
G
G
C
T
A
T
T
G
T
A
C
A
G
G
A
T
T
C
T
G
A
G
C
T
A
C
T
A
G
C
T
A
C
T
C
G
G
G
G
C
Glycine max PDS Glyma11G253000 CDS.seq
T
C
T
G
G
G
A
A
G
C
T
T
T
G
T
G
C
A
C
A
G
G
C
T
A
T
T
G
T
A
C
A
G
G
A
T
T
C
T
G
A
G
C
T
A
C
T
A
G
C
T
A
C
T
C
G
G
G
G
C
Glycine max PDS Glyma18G003900 CDS.seq
1,690
1,700
1,710
Ruler 1
C
A
G
A
A
A
A
G
A
A
T
G
G
C
T
A
A
A
G
C
A
A
G
T
G
T
T
G
T
G
T
A
A
Glycine max PDS Glyma11G253000 CDS.seq
C
A
G
A
A
A
A
G
A
A
T
G
G
C
T
A
A
A
G
C
A
A
G
T
G
T
T
G
T
G
T
A
A
Glycine max PDS Glyma18G003900 CDS.seq
GmPDS8 gRNA
GmPDS1 gRNA
GmPDS7 gRNA
GmPDS9 gRNA
GmPDS3 gRNA
B
Figure S1. Alignment of GmPDS11g and GmPDS18g nucleotide coding sequences. (A) Sequence alignment using ClustalW method. Non-conserved nucleotides are highlighted. The 20-nt guide RNA sequences are indicated in the purple boxes. (B) Comparison between GmPDS11g and GmPDS18g nucleotide coding sequences. The number in the upper-right cell indicates % identity, the number in the lower-left cell indicates distance.
