# Supplementary material for: An efficient and specific CRISPR-Cas9 genome editing system targeting soybean phytoene desaturase genes
Source: BMC Biotechnol. 2022 Feb 15;22:7. doi: 10.1186/s12896-022-00737-7 (PMC8845245; doi:10.1186/s12896-022-00737-7)
Supplement: Supplementary file 2 — Additional file 2. Figure S2. Alignment of GmPDS11g and GmPDS18g peptide sequences. [file 12896_2022_737_MOESM2_ESM.pptx]

## Slide 1
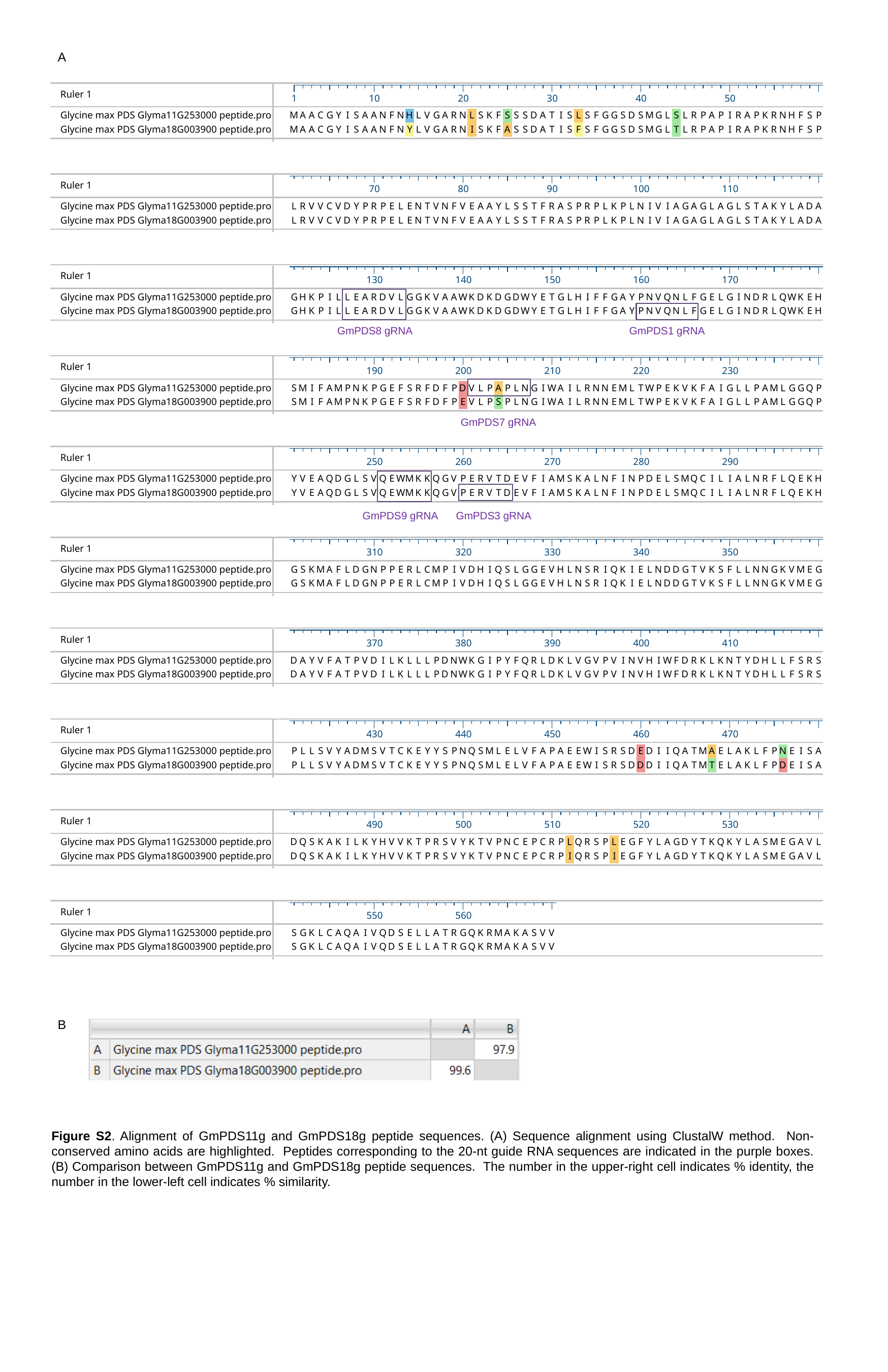

A
1
10
20
30
40
50
Ruler 1
M
A
A
C
G
Y
I
S
A
A
N
F
N
H
L
V
G
A
R
N
L
S
K
F
S
S
S
D
A
T
I
S
L
S
F
G
G
S
D
S
M
G
L
S
L
R
P
A
P
I
R
A
P
K
R
N
H
F
S
P
Glycine max PDS Glyma11G253000 peptide.pro
M
A
A
C
G
Y
I
S
A
A
N
F
N
Y
L
V
G
A
R
N
I
S
K
F
A
S
S
D
A
T
I
S
F
S
F
G
G
S
D
S
M
G
L
T
L
R
P
A
P
I
R
A
P
K
R
N
H
F
S
P
Glycine max PDS Glyma18G003900 peptide.pro
70
80
90
100
110
Ruler 1
L
R
V
V
C
V
D
Y
P
R
P
E
L
E
N
T
V
N
F
V
E
A
A
Y
L
S
S
T
F
R
A
S
P
R
P
L
K
P
L
N
I
V
I
A
G
A
G
L
A
G
L
S
T
A
K
Y
L
A
D
A
Glycine max PDS Glyma11G253000 peptide.pro
L
R
V
V
C
V
D
Y
P
R
P
E
L
E
N
T
V
N
F
V
E
A
A
Y
L
S
S
T
F
R
A
S
P
R
P
L
K
P
L
N
I
V
I
A
G
A
G
L
A
G
L
S
T
A
K
Y
L
A
D
A
Glycine max PDS Glyma18G003900 peptide.pro
130
140
150
160
170
Ruler 1
G
H
K
P
I
L
L
E
A
R
D
V
L
G
G
K
V
A
A
W
K
D
K
D
G
D
W
Y
E
T
G
L
H
I
F
F
G
A
Y
P
N
V
Q
N
L
F
G
E
L
G
I
N
D
R
L
Q
W
K
E
H
Glycine max PDS Glyma11G253000 peptide.pro
G
H
K
P
I
L
L
E
A
R
D
V
L
G
G
K
V
A
A
W
K
D
K
D
G
D
W
Y
E
T
G
L
H
I
F
F
G
A
Y
P
N
V
Q
N
L
F
G
E
L
G
I
N
D
R
L
Q
W
K
E
H
Glycine max PDS Glyma18G003900 peptide.pro
190
200
210
220
230
Ruler 1
S
M
I
F
A
M
P
N
K
P
G
E
F
S
R
F
D
F
P
D
V
L
P
A
P
L
N
G
I
W
A
I
L
R
N
N
E
M
L
T
W
P
E
K
V
K
F
A
I
G
L
L
P
A
M
L
G
G
Q
P
Glycine max PDS Glyma11G253000 peptide.pro
S
M
I
F
A
M
P
N
K
P
G
E
F
S
R
F
D
F
P
E
V
L
P
S
P
L
N
G
I
W
A
I
L
R
N
N
E
M
L
T
W
P
E
K
V
K
F
A
I
G
L
L
P
A
M
L
G
G
Q
P
Glycine max PDS Glyma18G003900 peptide.pro
250
260
270
280
290
Ruler 1
Y
V
E
A
Q
D
G
L
S
V
Q
E
W
M
K
K
Q
G
V
P
E
R
V
T
D
E
V
F
I
A
M
S
K
A
L
N
F
I
N
P
D
E
L
S
M
Q
C
I
L
I
A
L
N
R
F
L
Q
E
K
H
Glycine max PDS Glyma11G253000 peptide.pro
Y
V
E
A
Q
D
G
L
S
V
Q
E
W
M
K
K
Q
G
V
P
E
R
V
T
D
E
V
F
I
A
M
S
K
A
L
N
F
I
N
P
D
E
L
S
M
Q
C
I
L
I
A
L
N
R
F
L
Q
E
K
H
Glycine max PDS Glyma18G003900 peptide.pro
310
320
330
340
350
Ruler 1
G
S
K
M
A
F
L
D
G
N
P
P
E
R
L
C
M
P
I
V
D
H
I
Q
S
L
G
G
E
V
H
L
N
S
R
I
Q
K
I
E
L
N
D
D
G
T
V
K
S
F
L
L
N
N
G
K
V
M
E
G
Glycine max PDS Glyma11G253000 peptide.pro
G
S
K
M
A
F
L
D
G
N
P
P
E
R
L
C
M
P
I
V
D
H
I
Q
S
L
G
G
E
V
H
L
N
S
R
I
Q
K
I
E
L
N
D
D
G
T
V
K
S
F
L
L
N
N
G
K
V
M
E
G
Glycine max PDS Glyma18G003900 peptide.pro
370
380
390
400
410
Ruler 1
D
A
Y
V
F
A
T
P
V
D
I
L
K
L
L
L
P
D
N
W
K
G
I
P
Y
F
Q
R
L
D
K
L
V
G
V
P
V
I
N
V
H
I
W
F
D
R
K
L
K
N
T
Y
D
H
L
L
F
S
R
S
Glycine max PDS Glyma11G253000 peptide.pro
D
A
Y
V
F
A
T
P
V
D
I
L
K
L
L
L
P
D
N
W
K
G
I
P
Y
F
Q
R
L
D
K
L
V
G
V
P
V
I
N
V
H
I
W
F
D
R
K
L
K
N
T
Y
D
H
L
L
F
S
R
S
Glycine max PDS Glyma18G003900 peptide.pro
430
440
450
460
470
Ruler 1
P
L
L
S
V
Y
A
D
M
S
V
T
C
K
E
Y
Y
S
P
N
Q
S
M
L
E
L
V
F
A
P
A
E
E
W
I
S
R
S
D
E
D
I
I
Q
A
T
M
A
E
L
A
K
L
F
P
N
E
I
S
A
Glycine max PDS Glyma11G253000 peptide.pro
P
L
L
S
V
Y
A
D
M
S
V
T
C
K
E
Y
Y
S
P
N
Q
S
M
L
E
L
V
F
A
P
A
E
E
W
I
S
R
S
D
D
D
I
I
Q
A
T
M
T
E
L
A
K
L
F
P
D
E
I
S
A
Glycine max PDS Glyma18G003900 peptide.pro
490
500
510
520
530
Ruler 1
D
Q
S
K
A
K
I
L
K
Y
H
V
V
K
T
P
R
S
V
Y
K
T
V
P
N
C
E
P
C
R
P
L
Q
R
S
P
L
E
G
F
Y
L
A
G
D
Y
T
K
Q
K
Y
L
A
S
M
E
G
A
V
L
Glycine max PDS Glyma11G253000 peptide.pro
D
Q
S
K
A
K
I
L
K
Y
H
V
V
K
T
P
R
S
V
Y
K
T
V
P
N
C
E
P
C
R
P
I
Q
R
S
P
I
E
G
F
Y
L
A
G
D
Y
T
K
Q
K
Y
L
A
S
M
E
G
A
V
L
Glycine max PDS Glyma18G003900 peptide.pro
550
560
Ruler 1
S
G
K
L
C
A
Q
A
I
V
Q
D
S
E
L
L
A
T
R
G
Q
K
R
M
A
K
A
S
V
V
Glycine max PDS Glyma11G253000 peptide.pro
S
G
K
L
C
A
Q
A
I
V
Q
D
S
E
L
L
A
T
R
G
Q
K
R
M
A
K
A
S
V
V
Glycine max PDS Glyma18G003900 peptide.pro
GmPDS8 gRNA
GmPDS1 gRNA
GmPDS7 gRNA
GmPDS9 gRNA
GmPDS3 gRNA
B
Figure S2. Alignment of GmPDS11g and GmPDS18g peptide sequences. (A) Sequence alignment using ClustalW method. Non-conserved amino acids are highlighted. Peptides corresponding to the 20-nt guide RNA sequences are indicated in the purple boxes. (B) Comparison between GmPDS11g and GmPDS18g peptide sequences. The number in the upper-right cell indicates % identity, the number in the lower-left cell indicates % similarity.
