# Supplementary material for: An efficient and specific CRISPR-Cas9 genome editing system targeting soybean phytoene desaturase genes
Source: BMC Biotechnol. 2022 Feb 15;22:7. doi: 10.1186/s12896-022-00737-7 (PMC8845245; doi:10.1186/s12896-022-00737-7)
Supplement: Supplementary file 8 — Additional file 8. Figure S3. Example agarose gels showing detection of transgene in GmPDS8 T1 plants. [file 12896_2022_737_MOESM8_ESM.pptx]

## Slide 1
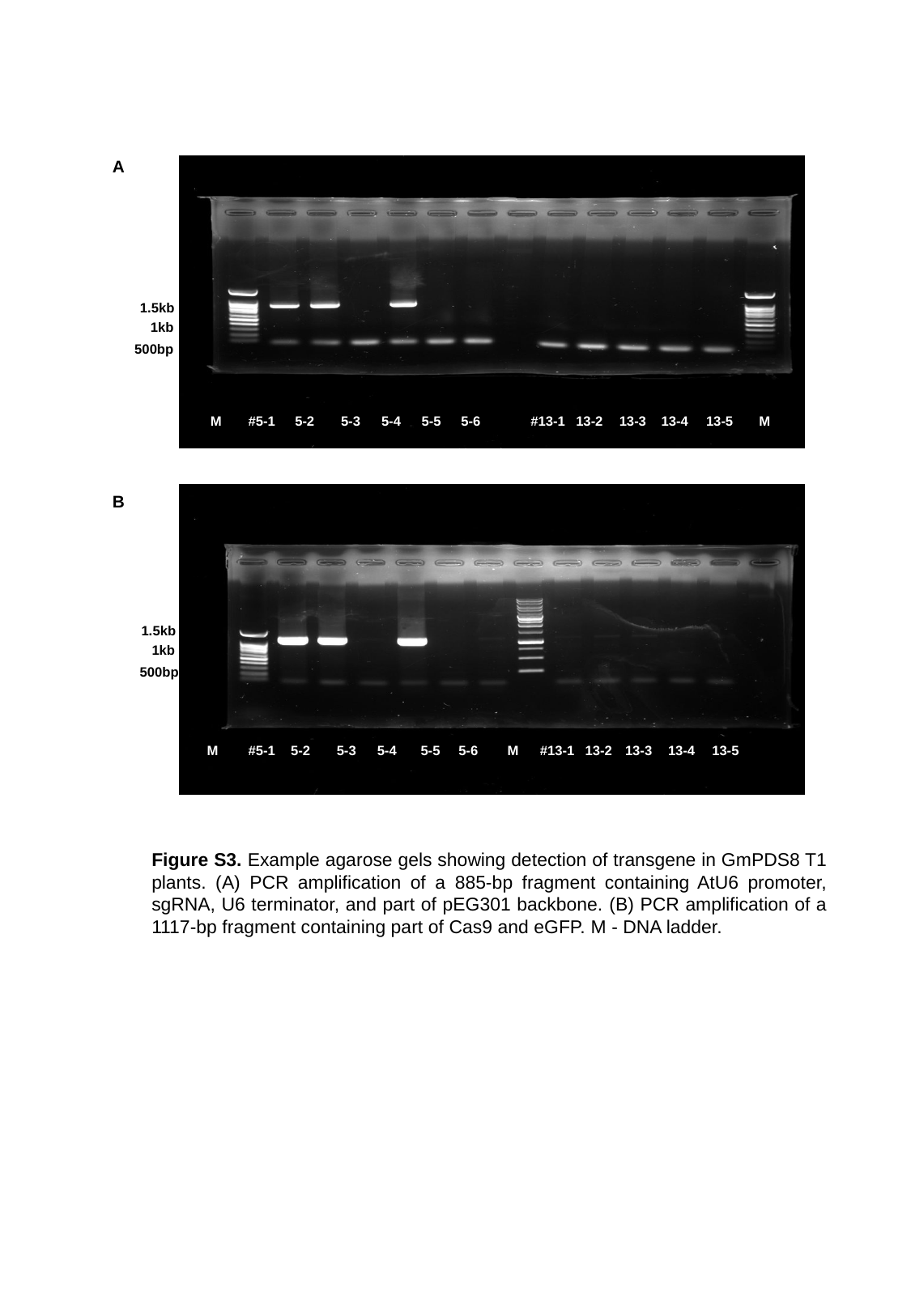

A
1.5kb
1kb
500bp
M
#5-1
5-2
5-3
5-4
5-5
5-6
#13-1
13-2
13-3
13-4
13-5
M
B
1.5kb
1kb
500bp
M
#5-1
5-2
5-3
5-4
5-5
5-6
M
#13-1
13-2
13-3
13-4
13-5
Figure S3. Example agarose gels showing detection of transgene in GmPDS8 T1 plants. (A) PCR amplification of a 885-bp fragment containing AtU6 promoter, sgRNA, U6 terminator, and part of pEG301 backbone. (B) PCR amplification of a 1117-bp fragment containing part of Cas9 and eGFP. M - DNA ladder.
